# Video game training and the reward system

**DOI:** 10.3389/fnhum.2015.00040

**Published:** 2015-02-05

**Authors:** Robert C. Lorenz, Tobias Gleich, Jürgen Gallinat, Simone Kühn

**Affiliations:** ^1^Department of Psychiatry and Psychotherapy, Charité-Universitätsmedizin Berlin, Campus MitteBerlin, Germany; ^2^Institute of Psychology, Humboldt-Universität zu BerlinBerlin, Germany; ^3^Department of Psychiatry and Psychotherapy, University Hospital Hamburg-EppendorfHamburg, Germany; ^4^Center for Lifespan Psychology, Max Planck Institute for Human DevelopmentBerlin, Germany

**Keywords:** video gaming, training, reward anticipation, longitudinal, fMRI

## Abstract

Video games contain elaborate reinforcement and reward schedules that have the potential to maximize motivation. Neuroimaging studies suggest that video games might have an influence on the reward system. However, it is not clear whether reward-related properties represent a precondition, which biases an individual toward playing video games, or if these changes are the result of playing video games. Therefore, we conducted a longitudinal study to explore reward-related functional predictors in relation to video gaming experience as well as functional changes in the brain in response to video game training. Fifty healthy participants were randomly assigned to a video game training (TG) or control group (CG). Before and after training/control period, functional magnetic resonance imaging (fMRI) was conducted using a non-video game related reward task. At pretest, both groups showed strongest activation in ventral striatum (VS) during reward anticipation. At posttest, the TG showed very similar VS activity compared to pretest. In the CG, the VS activity was significantly attenuated. This longitudinal study revealed that video game training may preserve reward responsiveness in the VS in a retest situation over time. We suggest that video games are able to keep striatal responses to reward flexible, a mechanism which might be of critical value for applications such as therapeutic cognitive training.

## INTRODUCTION

Over the last decades, the video gaming industry has grown into being one of the biggest multimedia industries in the world. Many people play video games on a day-to-day basis. For example in Germany 8 out of 10 people between 14 and 29 years of age reported to play video games, and 44% above age 29 still play video games. Taken together, based on survey data approximately more than 25 million people above 14 years of age (36%) play video games in Germany (Illek, 2013).

It seems as if human beings have a genuinely high motivation to play video games. Most frequently video games are played for the simple purpose of “fun” and a short-term increase in subjective well-being (Przybylski et al., 2010). Indeed, playing video games can satisfy different basic psychological needs, probably also dependent on the specific video game and its genre. Especially fulfillment of psychological needs like competence (sense of self-efficacy and acquisition of new skills), autonomy (personal goal-directed behavior in novel fictive environments), and relatedness (social interactions and comparisons) were associated with video gaming (Przybylski et al., 2010). Specifically, satisfaction of psychological needs might be mainly related to the various feedback mechanisms provided to the player by the game. This elaborate reinforcement and reward schedule has the potential to maximize motivation (Green and Bavelier, 2012).

Due to the high use, video games have come into the research focus of disciplines such as psychology and neuroscience. It has been shown that training with video games can lead to improvement in cognitive performance (Green and Bavelier, 2003, 2012; Basak et al., 2008), and in health-related behavior (Baranowski et al., 2008; Primack et al., 2012). Further, it has been shown that video games can be used in the training of surgeons (Boyle et al., 2011), that they are associated with higher psychological quality of life in elderly participants (Allaire et al., 2013; Keogh et al., 2013), and that they can facilitate weight reduction (Staiano et al., 2013). Although it is known that video games are designed to be maximally rewarding by game developers, and video gamers achieve psychological benefits from the gaming, the underlying processes that account for psychological benefits are not fully understood. Green and Bavelier (2012) concluded from their research that beyond the improvements in cognitive performance, the “true effect of action video game playing may be to enhance the ability to learn new tasks.” In other words, the effects of video game training might not be limited to the trained game itself; it may foster learning across a variety of tasks or domains. In fact, video game players learned how to learn new tasks quickly and therefore outperform non-video game players at least in the domain of attentional control (Green and Bavelier, 2012).

The underlying neurobiological processes associated with video gaming have been investigated with different imaging techniques and experimental designs. A raclopride positron emission tomography (PET) study by Koepp et al. (1998) showed that video gaming (more specifically, a tank simulation) is associated with endogenous dopamine release in the ventral striatum (VS). Furthermore, the level of dopamine binding potential has been related to performance in the game (Koepp et al., 1998). The VS is part of the dopaminergic pathways and is associated with reward processing and motivation (Knutson and Greer, 2008) as well as acquisition of learning in terms of prediction error signal (O’Doherty et al., 2004; Atallah et al., 2006; Erickson et al., 2010). Using magnetic resonance imaging (MRI) to measure gray matter volume, Erickson et al. (2010) showed that ventral and dorsal striatal volume could predict the early performance gains in a cognitively demanding video game (in particular, a two dimensional space shooter simulation). Additionally, Kühn et al. (2011) found that on the one hand frequent compared to infrequent video game playing was associated with higher structural gray matter volume and on the other hand was related to stronger functional activation during loss processing (Kühn et al., 2011). Further, striatal functional magnetic resonance imaging (fMRI) activity during actively playing or passively watching a video game (space shooter simulation, Erickson et al., 2010) or during completing a different non-video game related task (in particular an oddball task) predicted the subsequent training improvement (Vo et al., 2011). Taken together, these studies show that neural processes that are associated with video gaming are likely to be related to alterations of the neural processing in the VS, the core area of reward processing. Moreover, video gaming seems to be associated with structural and reward processing related functional changes in this area. However, it is not clear whether video game related structural and functional properties observed in earlier studies represent a *precondition*, which biases an individual toward playing video games or if these changes are the *result* of playing video games.

In summary, video games are quite popular and frequently used. One reason for that might be that video gaming may fulfill general human needs (Przybylski et al., 2010). Satisfied needs increase psychological well-being, which in turn is probably experienced as rewarding. Neuroimaging studies support this view by showing that video gaming is associated with alterations in the striatal reward system. Reward processing on the other hand is an essential mechanism for any human stimulus-response learning process. Green and Bavelier (2012) described video game training as a training for learning how to learn (learning of stimulus-response patterns is crucial to complete a video game successfully). We believe that video game training targets the striatal reward system (amongst other areas) and may lead to changes in reward processing. Therefore, in this study, we focus on striatal reward processing before and after video game training.

Here, we conducted a longitudinal study to be able to explore reward-related functional predictors in relation to performance and experience in the game as well as functional changes in the brain in response to video game training. We used a successful commercial video game, because commercial games are specifically designed to increase subjective well being (Ryan et al., 2006) and therefore game enjoyment and experienced reward during the game may be maximized. According to the prediction hypothesis, we expect that ventral striatal response in a reward task before video game training predicts performance as already shown in a previous study with a different task (Vo et al., 2011). Furthermore, we want to explore whether ventral striatal reward responsiveness is related to experienced fun, desire, or frustration in the training group during the training episode. To investigate the effect of video game training, we conducted a second MRI scan after video game training had taken place. Based on the findings by Kühn et al. (2011) showing altered reward processing in frequent compared to infrequent video game players, we expected altered striatal reward signal during reward anticipation in participants that had received training compared to controls. If there are functional changes in the striatal reward system, these should be related to the effect of video game training. If not, the observed changes in the study by Kühn et al. (2011) may rather relate to a precondition of the frequent video game players.

## MATERIALS AND METHODS

### PARTICIPANTS

Fifty healthy young adults were recruited via newspaper and internet advertisements and randomly assigned to video game training group (TG) or control group (CG). Preferably, we recruited only participants that played little or no video games in the last 6 months. None of the participants reported to play video games more than 1 h per week in the last 6 months (on average 0.7 h per month, SD = 1.97) and never played the training game [“Super Mario 64 (DS)”] before. Furthermore, the participants were free of mental disorders (according to personal interview using Mini-International Neuropsychiatric Interview), right-handed, and suitable for the MRI scanning procedure. The study was approved by the local Ethics Committee of the Charité – Universitätsmedizin Berlin and written informed consent was obtained from all participants after participants were fully instructed on the procedures of the study. Data of anatomical gray matter maps of these participants have been previously published (Kühn et al., 2013).

### TRAINING PROCEDURE

The TG (*n* = 25, mean age = 23.8 years, SD = 3.9 years, 18 females) was instructed to play “Super Mario 64 DS” on the “Nintendo Dual-Screen (DS) XXL” handheld console for at least 30 min per day over a period of 2 months. This extremely successful platformer game was chosen based on its high accessibility for video gaming naïve participants, as it offers a well-suited balance between reward delivery and difficulty and is popular among male and female participants. In the game, the player has to navigate through a complex 3D environment using buttons attached to the console used for movement, jumping, carrying, hitting, flying, stomping, reading, and character specific actions. Prior to the training, participants were instructed on general control and game mechanisms in a standardized way. During the training period, we offered different types of support (telephone, email, etc.) in case frustration or difficulties during game play arose.

The no-contact CG (*n* = 25, mean age = 23.4 years, SD = 3.7 years, 18 females) had no task in particular but underwent the same scanning procedure as the TG. All participants completed an fMRI scan at the beginning of the study (pretest) and 2 months after training or after a passive delay phase (posttest). The video game training for the TG began immediately after the pretest measurement and ended before posttest measurement.

### QUESTIONNAIRES

During training, the participants of the TG were asked to record the amount of daily gaming time. Furthermore participants rated experienced fun, frustration and desire to play during video gaming on a 7-point Likert scale once a week in a word processing document (see, supplementary material for more details) and sent the electronic data files via email to the experimenters. The accomplished game-related reward (stars collected) was objectively assessed by checking the video gaming console after training period. The maximum absolute amount of stars was 150.

### SLOT MACHINE PARADIGM

To investigate reward anticipation, a slightly modified slot machine paradigm was used that evoked strong striatal response (Lorenz et al., 2014). Participants had to go through the same slot machine paradigm before and after video game training procedure had taken place. The slot machine was programmed using Presentation software (Version 14.9, Neurobehavioral Systems Inc., Albany, CA, USA) and consisted of three wheels displaying two different sets of fruits (alternating fruit X and Y). At the two time points of measurement, a slot machine with cherries (X) and lemons (Y) or melons (X) and bananas (Y) were displayed in a counterbalanced fashion and equally distributed for the TG and CG. The color of two horizontal bars (above and below the slot machine) indicated the commands to start and stop the machine.

**FIGURE 1:**
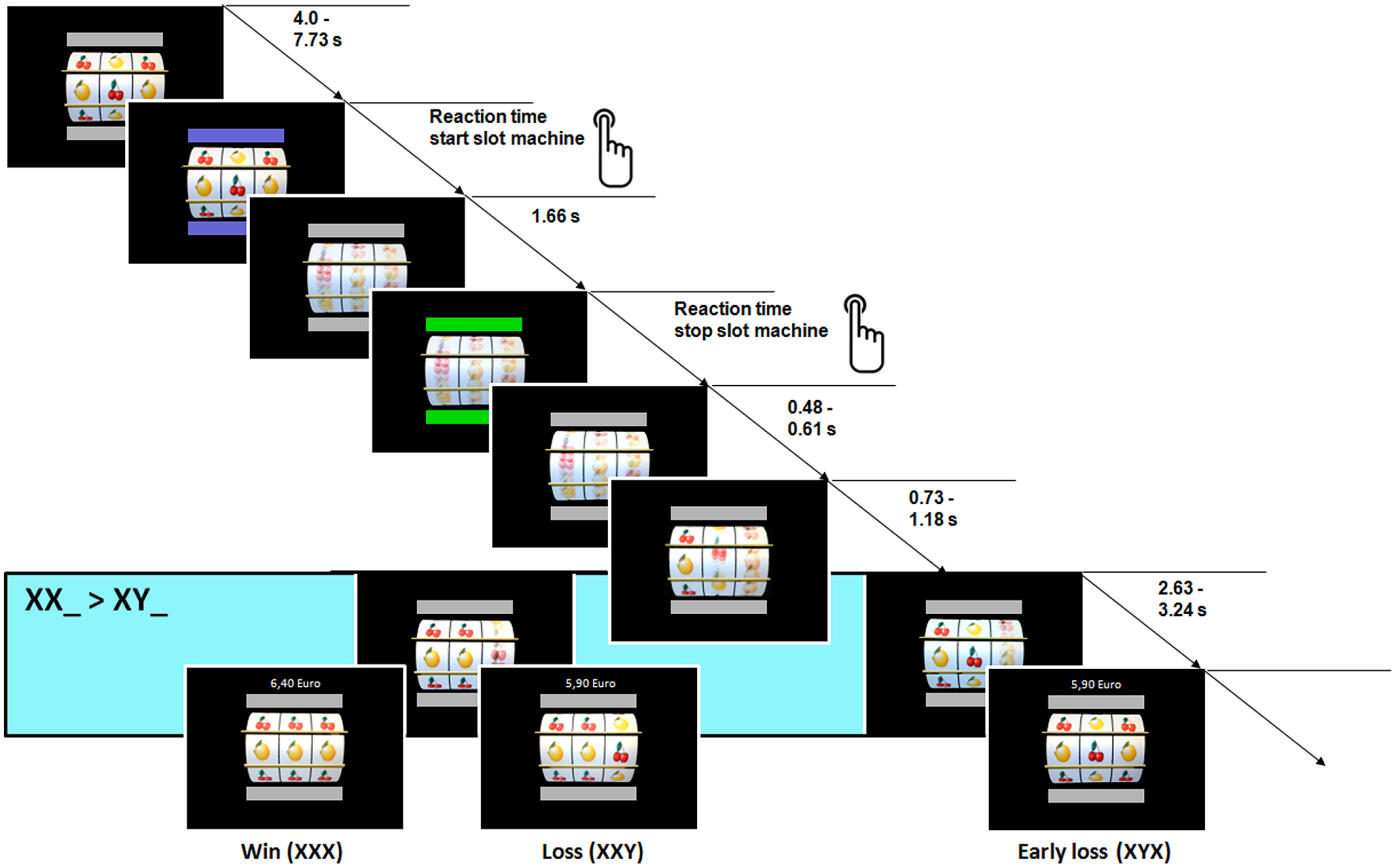
**Structure of the slot machine task.** FMRI analysis focused on stop of 2^nd^ wheel, when the first two wheels display the same fruit (XX_) or when the first two wheels displayed different fruits (XY_) while the 3^nd^ wheel was still rotating.

At the beginning of each trial, the wheels did not move and gray bars indicated the inactive state. When these bars turned blue (indicating the start of a trial), the participant was instructed to start the machine by pressing a button with the right hand. After a button press, the bars turned gray again (inactive state) and the three wheels started to rotate vertically with different accelerations (exponential increasing from left to right wheel, respectively). When the maximum rotation velocity of the wheels was reached (1.66 s after button press) the color of the bars turned green. This color change indicated that the participant could stop the machine by pressing the button again. After another button press, the three wheels successively stopped rotating from the left to the right side. The left wheel stopped after a variable delay of 0.48 and 0.61 s after the button press, while the middle and right wheel were still rotating. The second wheel stopped after an additional variable delay of 0.73 and 1.18 s. The right wheel stopped rotating after the middle wheel with a variable delay of 2.63 and 3.24 s. The stop of the third wheel terminated the trial and a feedback about the current win and the total amount of reward was displayed on the screen. For the next trial, the button changed from gray to blue again and the next trial started after a variable delay that ranged between 4.0 and 7.73 s and was characterized by an exponential decreasing function (see **Figure 1**).

The experiment contained 60 trials in total. The slot machine was determined with a pseudo-randomized distribution of 20 win trials (XXX or YYY), 20 loss trials (XXY or YYX), and 20 early loss trials (XYX, YXY, XYY, or YXX). Participants started with an amount of 6.00 euro representing the wager of 0.10 euro per trial (60 trials ∗ 0.10 euro wager = 6.00 euro wager) and gained 0.50 euro per trial, when all fruits in a row were of the same identity (XXX or YYY); if not, participants did not win (XXY, YYX, XYX, YXY, XYY, YYX) and the wager was subtracted from the total amount of money. Participants had no influence on winning or losing and the participants won the fixed amount of 10.00 euro (0.50 euro gain ∗ 20 win trials = 10.00 euro gain) at the end of the task. The participants were instructed to play the slot machine 60 times and that the aim in each trial is to get three fruit of the same kind in a row. Further, participants practiced the slot machine task before entering the scanner for 3–5 trials. No information was given that the task was a game of chance or any skill was involved.

### SCANNING PROCEDURE

Magnetic resonance imaging scans were conducted on a three Tesla Siemens TIM Trio Scanner (Siemens Healthcare, Erlangen, Germany), equipped with a 12 channel phased array head coil. Via a video projector, the slot machine paradigm was visually presented via a mirror system mounted on top of the head coil. Functional images were recorded using axial aligned T2^∗^-weighted gradient echo planar imaging (EPI) with the following parameters: 36 slices, interleaved ascending slice order, time to repeat (TR) = 2 s, time to echo (TE) = 30 ms, field of view (FoV) = 216 × 216, flip angle = 80°, voxel size: 3 mm × 3 mm × 3.6 mm. For anatomical reference, 3D anatomical whole brain images were obtained by a three-dimensional T1-weighted magnetization prepared gradient-echo sequence (MPRAGE; TR = 2500 ms; TE = 4.77 ms; inversion time = 1100 ms, acquisition matrix = 256 × 256 × 176, flip angle = 7°, voxel size: 1 mm × 1 mm × 1 mm).

### DATA ANALYSIS

#### Image processing

Magnetic resonance imaging data was analyzed using Statistical Parametric Mapping software package (SPM8, Wellcome Department of Imaging Neuroscience, London, UK). EPIs were corrected for acquisition time delay and head motion and then transformed into the stereotactic normalized standard space of Montreal Neuroimaging Institute using the unified segmentation algorithm as implemented in SPM8. Finally, EPIs were resampled (voxel size = 3 mm × 3 mm × 3 mm) and spatially smoothed with a 3D Gaussian kernel of 7 mm full width at half maximum.

#### Statistical analysis

A two-stage mixed-effects general linear model (GLM) was conducted. On single subject level, the model contained the data of both fMRI measurements, which was realized by fitting the data in different sessions. This GLM included separate regressors per session for gain anticipation (XX_ and YY_) and no gain anticipation (XY_ and YX_) as well as the following regressors of no interest: gain (XXX and YYY), loss (XXY and YYX), early loss (XYX, XYY, YXY, and YXX), button presses (after bar changed to blue as well as green), visual flow (rotation of the wheels), and the six rigid body movement parameters. Differential contrast images for gain anticipation against no gain anticipation (XX_ vs. XY_) were calculated for pre- and posttest and taken to group level analysis. On the second level, these differential *T*-contrast images were entered into a flexible factorial analysis of variance (ANOVA) with the factors group (TG vs. CG) and time (pre- vs. posttest).

Whole brain effects were corrected for multiple comparisons using a Monte Carlo simulation based cluster size correction (AlphaSim, Song et al., 2011). One thousand Monte Carlo simulations revealed a corresponding alpha error probability of *p* < 0.05, when using a minimum cluster size 16 adjacent voxels with a statistical threshold of *p* < 0.001. According to a meta-analysis by Knutson and Greer (2008), activation differences during reward anticipation were expected in the VS. Based on this a priori hypothesis, we further reported *post hoc* analysis within this brain area using a region of interest (ROI) analysis. To this end, we used a literature-based ROI for the VS (Schubert et al., 2008). These ROIs were created by combining previous functional findings regarding reward processing (predominantly monetary incentive delay task articles) with anatomical limits to gray matter brain tissue. Detailed information about the calculation of the VS ROI is described in supplementary material. Furthermore, we conducted a control analysis with the extracted mean parameters from the primary auditory cortex, because this region should be independent from the experimental manipulation in the reward task. Therefore we used an anatomical ROI of the Heschl’s gyri as described in the Anatomic Labeling (AAL) brain atlas (Tzourio-Mazoyer et al., 2002).

## RESULTS

### PREDICTION-RELATED RESULTS (PRETEST)

#### Brain response during gain anticipation

**FIGURE 2:**
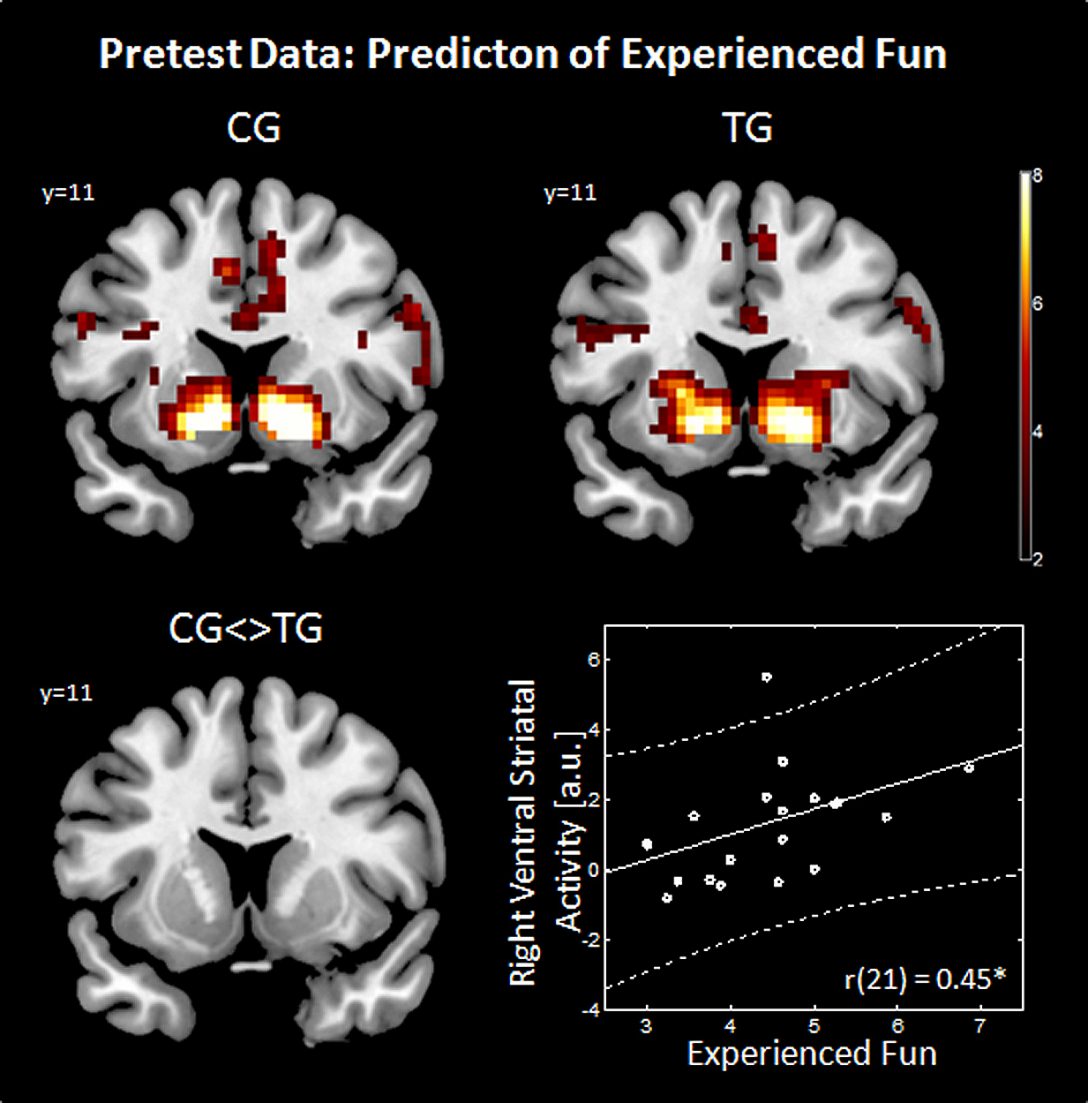
**Predictors of experienced fun.** The effect of gain anticipation (XX_) against no gain anticipation (XY_) is shown on a coronal slice (*Y* = 11) in the upper row for the control group (CG) and training group (TG). The group comparison (CG <> TG) is shown in the bottom left panel. Imaging results are threshold with *p* < 0.05, Monte Carlo corrected. Correlation between right ventral striatal activity (ROI extracted data) and experienced fun (average over weekly questionnaires) is shown in the bottom right panel. a.u., arbitrary units.

At pretest, during the slot machine task in both groups, gain anticipation (against no gain anticipation) evoked activation in a fronto-striatal network including subcortical areas (bilateral VS, thalamus), prefrontal areas (supplementary motor area, precentral gyrus, and middle frontal gyrus, superior frontal gyrus), and insular cortex. Additionally, increased activation in the occipital, parietal and temporal lobes was observed. All brain regions showing significant differences are listed in supplementary Tables S1 (for TG) and S2 (for CG). Note that the strongest activation differences were observed in the VS in both groups (see **Table 1**; **Figure 2**). For the contrast TG > CG, a stronger activation in the right supplementary motor area [SMA, cluster size 20 voxel, *T*(48) = 4.93, MNI-coordinates [x y z] = 9, 23, 49] and for CG > TG a stronger activation in the right pallidum (cluster size 20 voxel, *T*(48) = 5.66, MNI-coordinates [x y z] = 27, 8, 7) were observed. Both regions are probably not associated to reward-related functions as shown in the meta-analysis by Liu et al. (2011) across 142 reward studies.

**Table 1:** Group by time interaction (TG: Post > Pre) > (CG: Post > Pre) of the effect of gain anticipation against no gain anticipation in the whole brain analysis using Monte Carlo corrected significance threshold of *p* < 0.05. TG, training group; CG, control group; H, hemisphere; MNI, Montreal Neurological Institute; L, left; R, right.

Brain structure	H	Cluster size (vox)	Z (peak)	MNI coord. (mm)		
				*x*	*y*	*z*
Supplementary motor area	R	36	5.32	9	20	49
Insula lobe/inferior frontal gyrus (p. orbitalis)	L	23	4.81	-24	23	-2
Precentral gyrus	R	22	4.58	39	5	31
Ventral striatum	R	22	4.27	15	11	-8
Insula lobe/inferior frontal gyrus (p. orbitalis)	R	20	5.10	30	26	-8

#### Association between ventral striatal activity and associated video gaming behavior

To test the hypothesis of the predictive properties of striatal reward signal toward video games, the ventral striatal signal was individually extracted using the literature-based ROI and correlated with questionnaire items as well as game success, which was assessed by checking the video gaming console. Due to a lack of compliance of participants, weekly questionnaire data of four participants was missing. Weekly questions about experienced fun (*M* = 4.43, SD = 0.96), frustration (*M* = 3.8, SD = 1.03) and video gaming desire (*M* = 1.94, SD = 0.93) were averaged across the 2 months. Participants collected 87 stars (SD = 42.76) on average during the training period.

When applying Bonferroni correction to the calculated correlations (equal to a significance threshold of *p* < 0.006), none of the correlations were significant. Neither video gaming desire [left VS: *r*(21) = 0.03, *p* = 0.886; right VS: *r*(21) = -0.12, *p* = 0.614] nor frustration [left VS: *r*(21) = -0.24, *p* = 0.293; right VS: *r*(21) = -0.325, *p* = 0.15] nor accomplished game-related reward [left VS: *r*(25) = -0.17, *p* = 0.423; right VS: *r*(25) = -0.09, *p* = 0.685] were correlated with reward-related striatal activity. Interestingly, when using uncorrected significance threshold experienced fun during video gaming was correlated positively with the activity during gain anticipation in the right VS [*r*(21) = 0.45, *p* = 0.039] and a trend was observed in the left VS [*r*(21) = 0.37, *p* = 0.103] as shown in **Figure 2** (bottom right panel). However, when applying Bonferroni correction to this exploratory analysis, also the correlations between experienced fun and ventral striatal activity remained non-significant.

We further conducted a control analysis to investigate, whether this finding is specific for the VS. We correlated the same behavioral variables with the extracted parameter estimates of the Heschl’s gyri (primary auditory cortex). The analysis revealed no significant correlation (all *p*’s > 0.466).

### EFFECT OF VIDEO GAME TRAINING (PRE- AND POSTTEST)

**FIGURE 3:**
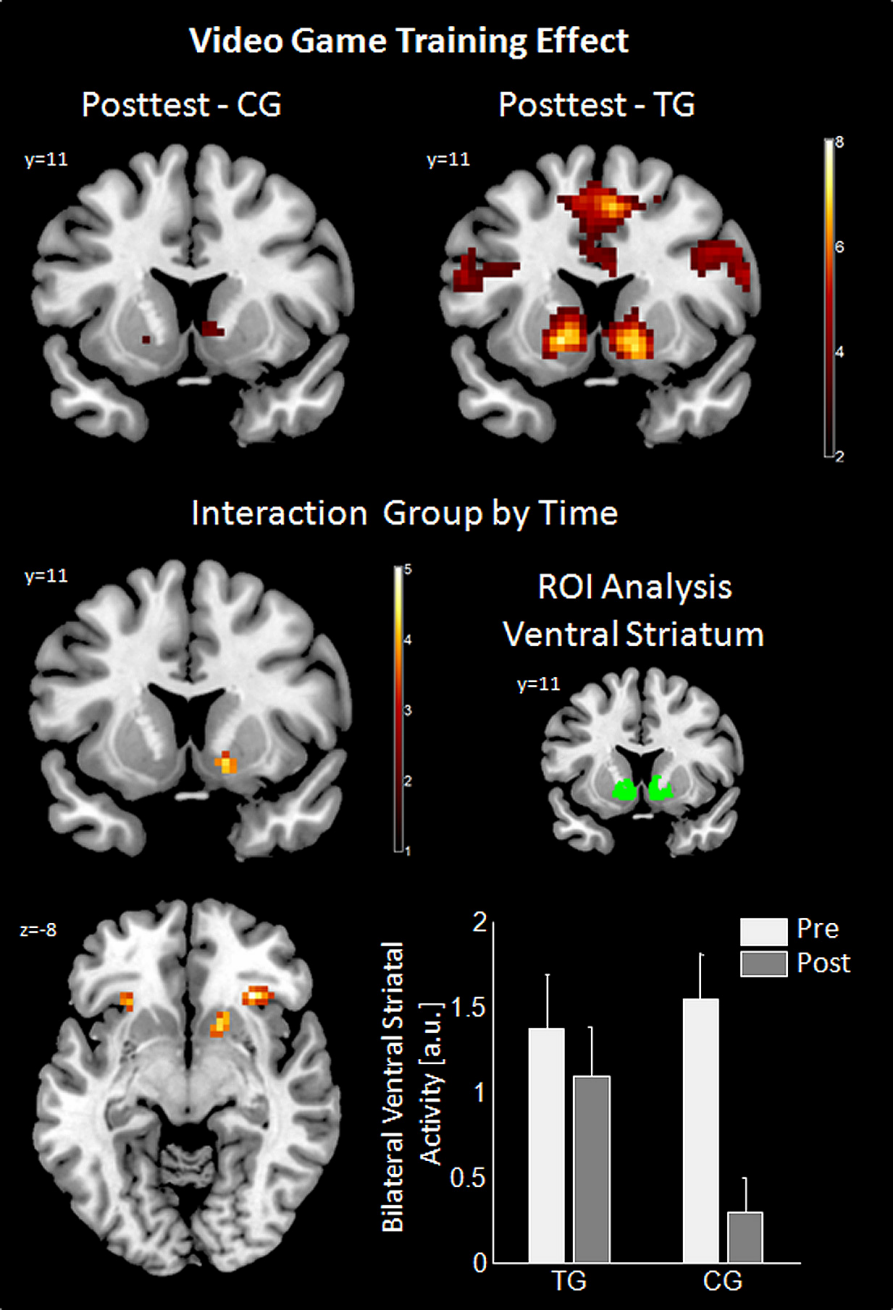
**Results of video game training effect.** For posttest the effect of gain anticipation (XX_) against no gain anticipation (XY_) is shown using a coronal cut (*Y* = 11) in the upper row for control group (CG) and training group (TG). Imaging results of the interaction group by time are shown in the middle and bottom left panel (axial cut at *Z* = -8). ROI analysis for this interaction is in the middle (literature-based ROI in green) and bottom (bar graph of the ROI analysis displayed with standard error of means) right panel. Imaging results are threshold with *p* < 0.05, Monte Carlo corrected. ROI, region of interest; a.u., arbitrary units.

Analysis of gain anticipation against no gain anticipation during the slot machine task at posttest revealed activation differences in the TG in the same fronto-striatal network as observed at pretest (for details see Table S3). In the CG, this effect was similar, but attenuated (see **Figure 3**; Table S4). The interaction effect of group by time revealed a significant difference in reward-related areas (right VS and bilateral insula/inferior frontal gyrus, pars orbitalis) and motor-related areas (right SMA and right precentral gyrus) indicating a preserved VS activity in the TG between the time points, but not in the CG. *Post hoc* ROI analysis using the literature-based VS ROI confirmed the interaction result [Interaction group by time: *F*(48,1) = 5.7, *p* = 0.021]. ROI-analysis in the control region (Heschl’s gyri) was non-significant. Additional *t*-tests revealed a significant difference between the time points within the CG group [*t*(24) = 4.6, *p* < 0.001] as well as a significant difference between the groups at posttest [*t*(48) = 2.27, *p* = 0.028]. Results for the interaction group by time are summarized in **Table 1** and are illustrated in **Figure 3**.

## DISCUSSION

The aim of the present study was twofold: We aimed at investigating how striatal reward responsiveness predicts video game related behavior and experience as well as the impact of video game training on functional aspects of the reward system. Regarding the prediction, we found a positive association between striatal reward signal at pretest and experienced fun during subsequent video game training. Regarding the effect of video gaming, a significant group by time interaction was observed driven by a decrease of the striatal reward signal in the CG.

### STRIATAL REWARD RESPONSIVENESS AND ITS PREDICTIVE PROPERTIES FOR VIDEO GAMING EXPERIENCE

A relationship between striatal reward signal and game performance or experienced desire and frustration was not observed. However, we were able to demonstrate a positive association of the striatal reward signal with experienced fun during video game training. Thus, we believe that the magnitude of striatal activity during reward processing in a non-video gaming related reward task is predictive for experienced fun during game play. However, this finding has to be interpreted with caution, since the observed correlation did not remain significant after correction for multiple testing.

A possible explanation for the correlation between striatal reward signal and experienced fun during video gaming might be that the measured striatal reward signal during slot machine gambling reflects the individuals’ reward responsiveness which may be associated with dopaminergic neurotransmission in the striatum. In accordance, previous studies showed that VS activity during reward anticipation is related to dopamine release in this region (Schott et al., 2008; Buckholtz et al., 2010). It has further been shown that also video gaming was associated with dopamine release in the same area (Koepp et al., 1998). Thus, the VS seems to be crucially involved in neural reward processing as well as video gaming, which involves many motivational and rewarding factors. Specifically, we are convinced that the observed relationship between VS activity and experienced fun might be related to a general responsiveness of the reward-related striatal dopamine system to hedonic stimuli. The VS has been associated with motivational and pleasure-elicited reactions in a recent review by Kringelbach and Berridge (2009). Thus, the observed association between ventral striatal activity and fun that refers to hedonic and pleasure-related experience during gaming seems well founded. Future studies should further investigate the relationship between striatal reward responsiveness and experienced fun during video gaming again to explore this relationship more deeply.

As mentioned above, striatal dopamine release (Koepp et al., 1998), volume (Erickson et al., 2010), and activity during gaming (Vo et al., 2011) were previously associated with video gaming performance. The results of the current study did not show an association between video gaming performance and VS activity. The achieved reward was operationalized by the number of accomplished missions/challenges in the game. Typical missions within the game are exemplified by defeating a boss, solving puzzles, finding secret places, racing an opponent, or gathering silver coins. These missions represent the progress in the game rather than the actual gaming performance. Thus, these variables may not be a sufficiently precise dependent variable of performance. We were, however, not able to collect more game-related variables, because “Super Mario 64 DS” is a commercial video game and a manipulation of this self-contained video game was impossible.

We further investigated the relationship between striatal reward signal and the experienced desire to play during video game training. Desire in this context is probably related to the need and expectations of video gaming’s potential satisfaction and reward. Desire is not clearly separable from wanting, because it usually arises together with wanting. Neurobiologically, wanting involves not only striatal, but also prefrontal areas that are related to goal-directed behavior (Cardinal et al., 2002; Berridge et al., 2010). Therefore, a neural correlate of desire might not be limited to the striatal reward area. Indeed, Kühn et al. (2013) showed that structural gray matter volume changes in the dorsolateral prefrontal cortex induced by video game training are positively associated with the subjective feeling of desire during video game training. Thus, in the current study the striatal reward responsiveness might not be related to desire, because desire might rather be associated with prefrontal goal-directed neural correlates. Future studies may investigate this in detail.

We expected a negative correlation between striatal reward responsiveness and experienced frustration during video game training since the VS activity is decreased at the omission of reward relative to the receipt of reward (Abler et al., 2005). However, this relationship was not observed. Previous studies showed that the insula is selectively activated in the context of frustration (Abler et al., 2005; Yu et al., 2014). Thus, future studies might also investigate insular activity in the context of omitted reward.

### EFFECT OF VIDEO GAME TRAINING ON THE REWARD SYSTEM

Kühn et al. (2011) showed in a cross-sectional study that frequent video game players (>9 h per week) demonstrated greater striatal reward-related activity compared to infrequent video game players. However, the question remained, whether this finding was a predisposition toward or a result of video gaming. In our present longitudinal study, gain anticipation during slot machine task revealed VS activity, which was preserved in TG over the 2 months, but not in CG. We assume that the striatal reward signal might reflect the motivational engagement during the slot machine task, which was still high in the TG at the posttest. The participants of the TG might preserve the responsiveness in reward processing and motivational willingness to complete the slot machine task at the second time point in a similarly engaged state as during the first time. An explanation for that finding might be that the video game training has an influence on dopamine-related reward processing during gaming (Koepp et al., 1998). Our results support this view, as this effect might temporally not be limited to the gaming session, but rather might have an influence on general striatal reward responsiveness in rewarding situations not related to video games. Kringelbach and Berridge (2009) showed that activity in the VS might represent an amplifier function of reward, and thus, video games might preserve reward responsiveness during game play itself, and even in the context of other rewarding tasks through amplification of pleasure-related activity. Thus, the video game training might be considered as an intervention targeting the dopaminergic neurotransmitter system, which might be investigated in the future. There is evidence, that dopaminergic interventions in the context of pharmacological studies can have a therapeutic behavior changing character. A recent pharmacological study using a dopaminergic intervention on older healthy adults by Chowdhury et al. (2013) showed that age-related impaired striatal reward processing signal could be restored by dopamine targeted drugs. Future studies should investigate the potential therapeutic effects of video gaming training on cognitive demanding tasks involving dopaminergic striatal signal. It would be highly valuable to uncover the specific effect of video gaming in the fronto-striatal circuitry. Our findings suggested an effect on reward processing, which in turn is essential for shaping of goal-directed behavior and flexible adaption to volatile environments (Cools, 2008). Therefore, tasks involving reward-related decisions such as reversal learning should be investigated in future longitudinal studies in combination with video game training. Multiple pharmacological studies have shown that a dopaminergic manipulation may lead to an increase or decrease in reversal learning performance, which probably depends on task demand and individual baseline dopamine levels (Klanker et al., 2013).

The observed effect of video game training on the reward system was also driven by a decrease in striatal activity in the CG during posttest, which may in part be explained by a motivational decline in the willingness to complete the slot machine task at the re-test. A study by Shao et al. (2013) demonstrated that even a single training session with a slot machine task before the actual scanning session led to decreases in striatal reward activity during win processing compared to a group that did not undergo a training session. A further study by Fliessbach et al. (2010) investigated the re-test reliability of three reward tasks and showed that the re-test reliability in VS during gain anticipation were rather poor, in contrast to motor-related reliabilities in primary motor cortex that were characterized as good. A possible explanation of these findings might be the nature of such reward tasks. The identical reward at both time points may not lead to the same reward signal at the second time of task performance, because the subjective reward feeling may be attenuated by a lack of novelty.

Obviously, in the present study the re-test was completed by both groups, but the decrease of the striatal reward activity was only observed in the CG, not in the TG. This preservation result in the TG may in part be related to the video game training as discussed above. Nevertheless, the CG was a no-contact group and did not complete an active control condition and thus, the findings might also represent a purely placebo like effect in the TG. However, even if not the specific video game training itself was the main reason for the preserved striatal response, our study may be interpreted as evidence arguing that video games lead to a rather strong placebo-like effect in a therapeutic or training-based setting. If video games would represent a stronger placebo effect than placebo medication or other placebo-like tasks is an open question. Moreover, during the scanning session itself participants were in the same situation in the scanner and one can expect that both groups produce the same social desirability effects. Still, the preservation effect should be interpreted very carefully, because placebo effect might confound the result (Boot et al., 2011). Future studies focusing on the reward system should include an active control condition in the study design.

Another possible limitation of the study might be that we did not control the video gaming behavior of the CG. We instructed the participants of the CG not to change their video gaming behavior in the waiting period and not to play Super Mario 64 (DS). However, video gaming behavior in the CG might have changed and could have affected the results. Future studies should include active control groups and assess video gaming behavior during the study period in detail.

In this study we focused on the VS. Nonetheless, we observed a significant training-related effect also in the insular cortices, SMA, and precentral gyrus. A recent meta-analysis by Liu et al. (2011) including 142 reward studies showed that besides the “core area of reward” VS also insula, ventromedial prefrontal cortex, anterior cingulate cortex, dorsolateral prefrontal cortex, and inferior parietal lobule are part of the reward network during reward anticipation. The insula is involved in the subjective integration of affective information, for instance during error-based learning in the context of emotional arousal and awareness (Craig, 2009; Singer et al., 2009). The activation during reward anticipation in the slot machine task may reflect subjective arousal and motivational involvement in the task. We believe that this significant training effect in the insula might – similar to the effect in VS – represent a motivational engagement, which was preserved in the TG at the posttest. Future studies could test this e.g., by applying arousal rating scales and correlate these values with insular activity. According to the differences in SMA and precentral gyrus, we want to highlight that these areas might not be involved in reward anticipation as it is not part of the suggested network of the mentioned meta-analysis (Liu et al., 2011). Instead, the SMA is involved in learning of motor-related stimulus-response associations among other functions (Nachev et al., 2008). With regard to the current study, SMA activity may reflect an updating process of the stimulus (slot machine with three rotating wheels) – response (button press to stop the slot machine) – consequence (here update of stop of the second wheel: XX_ and XY_) – chain. Speculatively, participants of the training group understand the slot machine after training as a video game, in which they could improve their performance by e.g., pressing the button at the right time point. In other words, the participants of the TG might have thought that they could impact the outcome of the slot machine by adapting their response pattern. Please note that the participants were not aware that the slot machine had a deterministic nature. As the precentral gyrus is also part of the motor system, the interpretation of the functional meaning of the SMA finding may be also valid for the precentral gyrus. Future studies might confirm these interpretations of SMA and precentral activation differences by systematically varying response-consequence-associations.

### VIDEO GAMING, SUPER MARIO, MOTIVATION, SUBJECTIVE WELL-BEING, AND THE REWARD SYSTEM

From a psychological view, joyful video games provide highly effective reward schedules, perfectly adjusted difficulty levels and strong engagement (Green and Bavelier, 2012). These specific properties potentially contain the opportunity to satisfy basic psychological needs such as competence, autonomy and relatedness (Przybylski et al., 2010). A study by Ryan et al. (2006) showed that participants feeling volitionally motivated by a 20 min training session of Super Mario 64 had an increased well-being after playing. This increased well-being was further associated with increases in the feeling of competence (e.g., experienced self-efficacy) and autonomy (e.g., acting based on interest). Together with the current finding of the preservation of the reward signal in a non-trained task, we believe that video games harbor the potential of a powerful tool for specific (cognitive) training. Depending on the video gaming genre and individual properties of the game, video games demand very complex cognitive and motor interactions from players to be able to reach the goal of the game and thus a specific training effect. The rewarding nature of video games may lead to a constant high motivational level within the training session.

## CONCLUSION

The current study showed that striatal reward responsiveness predicts the subsequent experienced video gaming fun suggesting that individual differences in reward responsiveness might affect motivational engagement of video gaming, but this interpretation needs confirmation in future studies. Furthermore, this longitudinal study revealed that video game training may preserve reward responsiveness in the VS in a re-test. We believe that video games are able to keep striatal responses to reward flexible, a mechanism which might be extremely important to keep motivation high, and thus might be of critical value for many different applications, including cognitive training and therapeutic possibilities. Future research should therefore investigate whether video game training might have an effect on reward-based decision-making, which is an important ability in everyday life.

## Conflict of Interest Statement

The authors declare that the research was conducted in the absence of any commercial or financial relationships that could be construed as a potential conflict of interest.
