# Supplementary material for: Video game training and the reward system
Source: Front Hum Neurosci. 2015 Feb 5;9:40. doi: 10.3389/fnhum.2015.00040 (PMC4318496; doi:10.3389/fnhum.2015.00040)
Supplement: Supplementary file 1 [file Data_Sheet_1.PDF]

Supplementary Material

Video game training and the reward system

Computation of literature based probabilistic region of interests

A Region of Interest (ROI) for the ventral striatum was created combining anatomical hypotheses with functional findings as reported in literature for comparable experimental designs. To this end, firstly we created an anatomical ROI for the basalganglia (as provided by the Automated Anatomical Labeling (AAL) brain atlas, Tzourio-Mazoyer et al., 2002). Secondly, spatial coordinates for this ROI were taken from fMRI publications from reward anticipation contrast of healthy volunteers (Abler et al., 2007, 2006; Bjork and Hommer, 2007; Bjork et al., 2010, 2004; Cooper and Knutson, 2008; Dichter et al., 2012; Dillon et al., 2008; Dreher et al., 2008; Elliott et al., 2000; Ernst et al., 2004; Figee et al., 2011; Galvan et al., 2007, 2005; Hoogman et al., 2011; Jones et al., 2011; Juckel et al., 2006; Kappel et al., 2013; Kirsch et al., 2003; Knutson and Greer, 2008; Knutson and Wimmer, 2007; Knutson et al., 2008, 2005, 2004, 2001a, 2001b; Kuhnen and Knutson, 2005; Martino et al., 2009; Ossewaarde et al., 2011; Samanez-Larkin et al., 2007; Schlagenhauf et al., 2008; Schmack et al., 2008; Spicer et al., 2007; Stoy et al., 2012; Ströhle et al., 2008; Wrase et al., 2007a, 2007b; Xue et al., 2010; Yau et al., 2012; Yu et al., 2010). Based on this data set, we created the ROI in a three-step process (Schubert et al., 2008):

(1) The probability that a voxel at a given position within an anatomical ROI showed neural activity regarding the corresponding literature was estimated by calculating a 3D normal (Gaussian) distribution G(x, y, z) as follows (Turkeltaub et al., 2002):

$$G(x,y,z) = \frac{1}{2\pi\sqrt{|Det(C)|}} \exp\left(-\frac{1}{2}\begin{bmatrix} x-\bar{x} & y-\bar{y} & z-\bar{z}\end{bmatrix}\right) C^{-1} \begin{bmatrix} x-\bar{x} \\ y-\bar{y} \\ z-\bar{z} \end{bmatrix}$$

where C is the covariance matrix for all coordinate triples x, y, z from the underlying literature and $\bar{x}$, $\bar{y}$, $\bar{z}$ are the mean values of the x, y, and z coordinates, respectively (Nielsen and Hansen, 2002).

(2) The outer limits of the finally used ROI were defined by the outer limits of the anatomical ROI and a threshold of two standard deviations of the resulting 3D distribution.

(3) Finally, a binary mask including all voxels within these boundaries was formed.

Note: The script for generating the probabilistic ROIs (written in Matlab by author TW and compatible with SPM8) and the full lists of coordinates used for ROI generation can be obtained from the authors upon request.

References

Abler, B., Erk, S., Walter, H., 2007. Human reward system activation is modulated by a single dose of olanzapine in healthy subjects in an event-related, double-blind, placebo-controlled fMRI study. Psychopharmacology (Berl.) 191, 823–833.

Abler, B., Walter, H., Erk, S., Kammerer, H., Spitzer, M., 2006. Prediction error as a linear function of reward probability is coded in human nucleus accumbens. NeuroImage 31, 790–795.

Bjork, J.M., Hommer, D.W., 2007. Anticipating instrumentally obtained and passively-received rewards: A factorial fMRI investigation. Behav. Brain Res. 177, 165–170.

Bjork, J.M., Knutson, B., Fong, G.W., Caggiano, D.M., Bennett, S.M., Hommer, D.W., 2004. Incentive-Elicited Brain Activation in Adolescents: Similarities and Differences from Young Adults. J. Neurosci. 24, 1793–1802.

Bjork, J.M., Smith, A.R., Chen, G., Hommer, D.W., 2010. Adolescents, Adults and Rewards: Comparing Motivational Neurocircuitry Recruitment Using fMRI. PLoS ONE 5, e11440.

Cooper, J.C., Knutson, B., 2008. Valence and salience contribute to nucleus accumbens activation. NeuroImage 39, 538–547.

Dichter, G.S., Felder, J.N., Green, S.R., Rittenberg, A.M., Sasson, N.J., Bodfish, J.W., 2012. Reward circuitry function in autism spectrum disorders. Soc. Cogn. Affect. Neurosci. 7, 160–172.

Dillon, D.G., Holmes, A.J., Jahn, A.L., Bogdan, R., Wald, L.L., Pizzagalli, D.A., 2008. Dissociation of neural regions associated with anticipatory versus consummatory phases of incentive processing. Psychophysiology 45, 36–49.

Dreher, J.-C., Meyer-Lindenberg, A., Kohn, P., Berman, K.F., 2008. Age-related changes in midbrain dopaminergic regulation of the human reward system. Proc. Natl. Acad. Sci. 105, 15106–15111.

Elliott, R., Friston, K.J., Dolan, R.J., 2000. Dissociable Neural Responses in Human Reward Systems. J. Neurosci. 20, 6159–6165.

Ernst, M., Nelson, E.E., McClure, E.B., Monk, C.S., Munson, S., Eshel, N., Zarahn, E., Leibenluft, E., Zametkin, A., Towbin, K., Blair, J., Charney, D., Pine, D.S., 2004.

Choice selection and reward anticipation: an fMRI study. Neuropsychologia 42, 1585–1597.

Figee, M., Vink, M., de Geus, F., Vulink, N., Veltman, D.J., Westenberg, H., Denys, D., 2011. Dysfunctional Reward Circuitry in Obsessive-Compulsive Disorder. Biol. Psychiatry 69, 867–874.

Galvan, A., Hare, T., Voss, H., Glover, G., Casey, B. j., 2007. Risk-taking and the adolescent brain: who is at risk? Dev. Sci. 10, F8–F14.

Galvan, A., Hare, T.A., Davidson, M., Spicer, J., Glover, G., Casey, B.J., 2005. The Role of Ventral Frontostriatal Circuitry in Reward-Based Learning in Humans. J. Neurosci. 25, 8650–8656.

Hoogman, M., Aarts, E., Zwiers, M., Slaats-Willemse, D., Naber, M., Onnink, M., Cools, R., Kan, C., Buitelaar, J., Franke, B., 2011. Nitric Oxide Synthase Genotype Modulation of Impulsivity and Ventral Striatal Activity in Adult ADHD Patients and Healthy Comparison Subjects. Am. J. Psychiatry 168, 1099–1106.

Jones, C.L., Minati, L., Harrison, N.A., Ward, J., Critchley, H.D., 2011. Under Pressure: Response Urgency Modulates Striatal and Insula Activity during Decision-Making under Risk. PLoS ONE 6, e20942.

Juckel, G., Schlagenhauf, F., Koslowski, M., Wüstenberg, T., Villringer, A., Knutson, B., Wrase, J., Heinz, A., 2006. Dysfunction of ventral striatal reward prediction in schizophrenia. NeuroImage 29, 409–416.

Kappel, V., Koch, A., Lorenz, R.C., Brühl, R., Renneberg, B., Lehmkuhl, U., Salbach-Andrae, H., Beck, A., 2013. CID: a valid incentive delay paradigm for children. J. Neural Transm. 120, 1259–1270.

Kirsch, P., Schienle, A., Stark, R., Sammer, G., Blecker, C., Walter, B., Ott, U., Burkart, J., Vaitl, D., 2003. Anticipation of reward in a nonaversive differential conditioning paradigm and the brain reward system:: an event-related fMRI study. NeuroImage 20, 1086–1095.

Knutson, B., Adams, C.M., Fong, G.W., Hommer, D., 2001a. Anticipation of increasing monetary reward selectively recruits nucleus accumbens. J. Neurosci. Off. J. Soc. Neurosci. 21, RC159.

Knutson, B., Bhanji, J.P., Cooney, R.E., Atlas, L.Y., Gotlib, I.H., 2008. Neural Responses to Monetary Incentives in Major Depression. Biol. Psychiatry 63, 686–692.

Knutson, B., Bjork, J.M., Fong, G.W., Hommer, D., Mattay, V.S., Weinberger, D.R., 2004. Amphetamine Modulates Human Incentive Processing. Neuron 43, 261–269.

Knutson, B., Fong, G.W., Adams, C.M., Varner, J.L., Hommer, D., 2001b. Dissociation of reward anticipation and outcome with event-related fMRI. Neuroreport 12, 3683–3687.

Knutson, B., Greer, S.M., 2008. Anticipatory affect: neural correlates and consequences for choice. Philos. Trans. R. Soc. B Biol. Sci. 363, 3771–3786.

Knutson, B., Taylor, J., Kaufman, M., Peterson, R., Glover, G., 2005. Distributed Neural Representation of Expected Value. J. Neurosci. 25, 4806–4812.

Knutson, B., Wimmer, G.E., 2007. Splitting the Difference. Ann. N. Y. Acad. Sci. 1104, 54–69.

Kuhnen, C.M., Knutson, B., 2005. The Neural Basis of Financial Risk Taking. Neuron 47, 763–770.

Martino, B.D., Kumaran, D., Holt, B., Dolan, R.J., 2009. The Neurobiology of Reference-Dependent Value Computation. J. Neurosci. 29, 3833–3842.

Nielsen, F.A., Hansen, L.A., 2002. Automatic anatomical labeling of Talairach coordinates and generation of volumes of interest via the BrainMap database. Neuroimage 16.

Ossewaarde, L., Verkes, R.J., Hermans, E.J., Kooijman, S.C., Urner, M., Tendolkar, I., van Wingen, G.A., Fernández, G., 2011. Two-Week Administration of the Combined Serotonin-Noradrenaline Reuptake Inhibitor Duloxetine Augments Functioning of Mesolimbic Incentive Processing Circuits. Biol. Psychiatry 70, 568–574.

Samanez-Larkin, G.R., Gibbs, S.E.B., Khanna, K., Nielsen, L., Carstensen, L.L., Knutson, B., 2007. Anticipation of monetary gain but not loss in healthy older adults. Nat. Neurosci. 10, 787–791.

Schlagenhauf, F., Juckel, G., Koslowski, M., Kahnt, T., Knutson, B., Dembler, T., Kienast, T., Gallinat, J., Wrase, J., Heinz, A., 2008. Reward system activation in schizophrenic patients switched from typical neuroleptics to olanzapine. Psychopharmacology (Berl.) 196, 673–684.

Schmack, K., Schlagenhauf, F., Sterzer, P., Wrase, J., Beck, A., Dembler, T., Kalus, P., Puls, I., Sander, T., Heinz, A., Gallinat, J., 2008. Catechol-O-methyltransferase val158met genotype influences neural processing of reward anticipation. NeuroImage 42, 1631–1638.

Schubert, R., Ritter, P., Wustenberg, T., Preuschhof, C., Curio, G., Sommer, W., Villringer, A., 2008. Spatial Attention Related SEP Amplitude Modulations Covary with BOLD Signal in S1-A Simultaneous EEG-fMRI Study. Cereb. Cortex 18, 2686–2700.

Spicer, J., Galvan, A., Hare, T.A., Voss, H., Glover, G., Casey, B., 2007. Sensitivity of the Nucleus Accumbens to Violations in Expectation of Reward. Neuroimage 34, 455–461.

Stoy, M., Schlagenhauf, F., Sterzer, P., Bermpohl, F., Hägele, C., Suchotzki, K., Schmack, K., Wrase, J., Ricken, R., Knutson, B., Adli, M., Bauer, M., Heinz, A., Ströhle, A., 2012. Hyporeactivity of ventral striatum towards incentive stimuli in unmedicated depressed

patients normalizes after treatment with escitalopram. J. Psychopharmacol. (Oxf.) 26, 677–688.

Ströhle, A., Stoy, M., Wrase, J., Schwarzer, S., Schlagenhauf, F., Huss, M., Hein, J., Nedderhut, A., Neumann, B., Gregor, A., Juckel, G., Knutson, B., Lehmkuhl, U., Bauer, M., Heinz, A., 2008. Reward anticipation and outcomes in adult males with attention-deficit/hyperactivity disorder. Neuroimage 39, 966–972.

Turkeltaub, P.E., Eden, G.F., Jones, K.M., Zeffiro, T.A., 2002. Meta-analysis of the functional neuroanatomy of single-word reading: Method and validation. Neuroimage 16, 765–780.

Tzourio-Mazoyer, N., Landeau, B., Papathanassiou, D., Crivello, F., Etard, O., Delcroix, N., Mazoyer, B., Joliot, M., 2002. Automated anatomical labeling of activations in SPM using a macroscopic anatomical parcellation of the MNI MRI single-subject brain. Neuroimage 15, 273–289.

Wrase, J., Kahnt, T., Schlagenhauf, F., Beck, A., Cohen, M.X., Knutson, B., Heinz, A., 2007a. Different neural systems adjust motor behavior in response to reward and punishment. Neuroimage 36, 1253–1262.

Wrase, J., Schlagenhauf, F., Kienast, T., Wüstenberg, T., Bermpohl, F., Kahnt, T., Beck, A., Ströhle, A., Juckel, G., Knutson, B., Heinz, A., 2007b. Dysfunction of reward processing correlates with alcohol craving in detoxified alcoholics. Neuroimage 35, 787–794.

Xue, G., Lu, Z., Levin, I.P., Bechara, A., 2010. The Impact of Prior Risk Experiences on Subsequent Risky Decision-Making: The Role of the Insula. Neuroimage 50, 709–716.

Yau, W.-Y.W., Zubieta, J.-K., Weiland, B.J., Samudra, P.G., Zucker, R.A., Heitzeg, M.M., 2012. Nucleus Accumbens Response to Incentive Stimuli Anticipation in Children of Alcoholics: Relationships with Precursive Behavioral Risk and Lifetime Alcohol Use. J. Neurosci. 32, 2544–2551.

Yu, R., Mobbs, D., Seymour, B., Calder, A.J., 2010. Insula and Striatum Mediate the Default Bias. J. Neurosci. 30, 14702–14707.

Items of the Questionnaire

We assessed experienced fun, frustration and desire to play during video gaming on a 7-point Likert scale once a week in a word processing document. Therefore we asked to rate statements from “not true” (=1) to “perfectly true” (=7) for experienced fun (“Playing the game is fun to me”) and experienced frustration (“I am frustrated by the game.”). Desire to play video games was rated by the request “Please rate your mean desire to play video games” from “nonexistent” (=1) to “very high” (=7).

Table S1: Effect of Gain anticipation against no gain anticipation for video game training group for pretest using Monte Carlo corrected significance threshold of $p<0.05$.

Brain structure	H	Cluster size (vox)	T (peak)	MNI coord. (mm)		
				x	y	z
Ventral Striatum	R	2040	8.91	15	11	-5
Ventral Striatum	L		8.88	-15	8	-8
Insula Lobe	L		8.20	-30	29	-2
Thalamus	R		7.70	6	-16	1
Insula Lobe	R		7.56	33	26	7
Superior Parietal Lobule	L	1140	8.39	-24	-52	58
Supra Marginal Gyrus	L		7.61	-51	-25	34
Postcentral Gyrus	L		7.46	-30	-40	58
Superior Occipital Gyrus	L		6.95	-24	-79	25
Inferior Parietal Lobule	L		6.01	-30	-40	40
Precentral Gyrus	L	978	9.75	-27	-10	52
	R		8.71	33	-7	52
Middle Frontal Gyrus	R		8.27	30	-4	55
SMA	R		8.14	9	5	52
Superior Frontal Gyrus	L		8.02	-21	-10	55
Middle Temporal Gyrus	R	313	6.98	48	-70	-2
Lingual Gyrus	R		6.12	27	-91	-8
Superior Parietal Lobule	R	249	6.99	18	-55	61
Postcentral Gyrus	R		6.50	33	-37	55
Inferior Parietal Lobule	R		5.57	30	-49	55
Cerebellum Vermis	R	172	6.17	3	-70	-32
Cerebellum Lobule VI	L		4.59	-9	-70	-23
Calcarine Gyrus	R	108	4.91	18	-82	4
Cuneus	R	103	7.09	21	-73	34
Precentral Gyrus	R	100	7.92	57	5	31
Anterior Cingulate Cortex	R	66	5.55	9	44	28
Postcentral Gyrus	R	59	4.97	51	-19	34
Fusiform Gyrus	R	57	4.52	39	-55	-17
Calcarine Gyrus	L	28	4.75	-15	-73	7

Table S2: Effect of Gain anticipation against no gain anticipation for control group for pretest using Monte Carlo corrected significance threshold of p<0.05.

Brain structure	H	Cluster size (vox)	T (peak)	MNI coord. (mm)		
				x	y	z
Ventral Striatum	L	1613	12.40	-9	8	-5
Ventral Striatum	R		11.41	15	11	-8
Insula Lobe / Inferior Frontal Gyrus (p. Orbitalis)	R		10.64	30	26	-8
Insula Lobe	L		10.52	-30	26	-5
Thalamus	R		6.58	6	-13	1
Thalamus	L		6.51	-6	-16	-2
Superior Frontal Gyrus	L	1312	9.27	-21	-4	55
Precentral Gyrus	L		9.25	-27	-10	52
Superior Frontal Gyrus	R		8.93	21	-7	58
SMA	R		8.19	9	5	52
Precentral Gyrus	R		7.31	39	-7	49
Superior Parietal Lobule	L	754	6.76	-21	-58	58
Supra Marginal Gyrus	L		6.46	-51	-25	34
Inferior Parietal Lobule	L		6.41	-21	-52	52
Superior Occipital Gyrus	L		5.80	-24	-76	22
Postcentral Gyrus	L		5.72	-54	-19	31
Superior Parietal Lobule	R	201	7.78	30	-49	58
Middle Temporal Gyrus	R	127	5.84	48	-70	1
Calcarine Gyrus	R	107	6.45	15	-79	4
Inferior Occipital Gyrus	R	101	5.57	27	-91	-11
Cerebellum Vermis	R	69	4.76	3	-73	-29
Lingual Gyrus	R	66	4.77	27	-55	-8
Superior Occipital Gyrus	R	63	5.60	27	-76	28
Middle Cingulate Cortex	L	57	6.18	-12	-19	43
Middle Cingulate Cortex	R	41	6.23	15	-22	40
Calcarine Gyrus	L	40	4.80	-12	-76	7
Inferior Occipital Gyrus	L	38	6.64	-21	-88	-11
Postcentral Gyrus	R	36	5.10	51	-19	31

Table S3: Effect of Gain anticipation against no gain anticipation for video game training group for posttest using Monte Carlo corrected significance threshold of p<0.05.

Brain structure	H	Cluster size (vox)	T (peak)	MNI coord. (mm)		
				x	y	z
Superior Occipital Gyrus	L	1366	7.67	-24	-79	25
Inferior Parietal Lobule	L		7.06	-27	-43	52
Middle Occipital Gyrus	L		6.91	-48	-70	1
Inferior Parietal Lobule	L		6.69	-48	-25	37
Superior Parietal Lobule	L		6.33	-21	-61	52
Calcarine Gyrus	L		6.21	-12	-100	-5
Postcentral Gyrus	L		5.88	-57	-19	28
Precentral Gyrus	L	1353	8.69	-27	-7	49
SMA	R		7.96	6	8	52
Middle Frontal Gyrus	R		7.56	36	-7	52
Precentral Gyrus	R		7.46	45	5	31
SMA	L		6.18	-6	-4	55
Superior Frontal Gyrus	L		5.53	-24	-4	61
Middle Temporal Gyrus	R	1222	8.05	48	-67	1
Superior Occipital Gyrus	R		6.52	21	-76	40
Middle Occipital Gyrus	R		6.10	30	-88	1
Superior Occipital Gyrus	R		6.04	27	-76	31
Fusiform Gyrus	R		5.58	30	-58	-14
Calcarine Gyrus	R		5.36	15	-79	4
Cuneus	R		5.25	18	-67	37
Inferior Occipital Gyrus	R		5.23	36	-85	-14
Ventral Striatum	R	599	6.99	15	11	-8
Thalamus	L		5.59	-6	-19	1
Thalamus	R		5.47	12	-13	-2
Putamen	L	222	7.60	-15	8	-5
Ventral Striatum	L		7.40	-15	11	-5
Pallidum	L		6.46	-18	2	-2
Superior Parietal Lobule	R	209	7.07	24	-55	58
Postcentral Gyrus	R		5.84	36	-37	55
Insula Lobe	R	194	6.31	30	29	-2
Inferior Frontal Gyrus (p. Orbitalis)	R		6.01	36	26	-8
Insula Lobe	R		4.96	36	17	7
Precentral Gyrus	L	170	5.96	-51	2	34
Inferior Frontal Gyrus (p. Opercularis)	L		5.44	-57	8	19
Insula Lobe	L	160	7.90	-27	26	-2
Inferior Frontal Gyrus (p. Orbitalis)	L		3.89	-33	20	-20

Cerebellum Vermis	R	122	5.48	0	-58	-38
Middle Cingulate Cortex	R	120	4.37	6	41	31
Anterior Cingulate Cortex	R		4.30	0	32	28
Superior Medial Gyrus	L		4.04	-6	44	34
Superior Temporal Gyrus	R	61	6.12	66	-37	13
Middle Temporal Gyrus	R		3.47	60	-46	4
Middle Temporal Gyrus	L	45	4.74	-51	-46	10
Brainstem	R	32	4.85	3	-37	-38
Middle Cingulate Cortex	R	29	4.78	6	-25	46
Middle Frontal Gyrus	L	28	4.25	-39	38	25
Postcentral Gyrus	R	26	4.64	60	-16	34
Calcarine Gyrus	L	23	4.98	-12	-79	7
Middle Cingulate Cortex	L	22	4.49	-9	-22	43
Insula Lobe	L	20	5.50	-39	-4	7
Hippocampus	L	20	4.10	-18	-28	-5
Inferior Temporal Gyrus	L	16	4.56	-42	-49	-17

Table S4: Effect of Gain anticipation against no gain anticipation for control group for posttest using Monte Carlo corrected significance threshold of $p<0.05$.

Brain structure	H	Cluster size (vox)	T (peak)	MNI coord. (mm)		
				x	y	z
Thalamus	L	200	5.25	-6	-22	1
Thalamus	R		4.42	9	-16	1
Ventral Striatum	R	147	5.59	6	5	-2
Ventral Striatum	L		5.16	-9	5	-5
Caudate Nucleus	L		5.15	-6	5	7
Pallidum	L		4.38	-18	2	-2
Pallidum	R		4.38	15	5	-5
Precentral Gyrus	R	130	4.85	39	-7	46
Middle Frontal Gyrus	R		4.84	39	-7	58
Superior Frontal Gyrus	R		4.49	24	-7	58
Superior Occipital Gyrus	R	99	5.22	27	-76	31
Precentral Gyrus	L	93	5.50	-33	-7	46
Superior Frontal Gyrus	L		5.48	-21	-1	52
Precentral Gyrus	L		5.13	-39	-7	46
Calcarine Gyrus	R	84	5.19	15	-79	4
Lingual Gyrus	R		4.62	21	-91	-5
Superior Parietal Lobule	R	75	5.50	24	-55	58
Postcentral Gyrus	R		4.59	30	-34	46
Postcentral Gyrus	R		4.47	33	-34	52
Middle Temporal Gyrus	R	60	5.01	45	-58	-2
Middle Occipital Gyrus	R		4.50	42	-67	4
Insula Lobe	L	57	6.56	-30	29	-2
Superior Occipital Gyrus	L	52	4.68	-24	-79	25
Inferior Occipital Gyrus	L	48	5.86	-21	-88	-8
Lingual Gyrus	L		4.56	-12	-94	-11
SMA	R	30	6.76	9	8	52
Anterior Cingulate Cortex	R/L	27	4.29	0	5	28
Insula Lobe	R	24	4.00	33	26	1
Precentral Gyrus	L	22	4.40	-51	2	31
Postcentral Gyrus	R	20	4.96	51	-16	31

Middle Occipital Gyrus	R	20	3.89	33	-85	13
